# Novel Pentablock Copolymers as Thermosensitive Self-Assembling Micelles for Ocular Drug Delivery

**DOI:** 10.15171/apb.2017.003

**Published:** 2017-04-13

**Authors:** Mitra Alami-Milani, Parvin Zakeri-Milani, Hadi Valizadeh, Roya Salehi, Sara Salatin, Ali Naderinia, Mitra Jelvehgari

**Affiliations:** ^1^Department of Pharmaceutics, Faculty of Pharmacy, Tabriz University of Medical Sciences, Tabriz, Iran.; ^2^Student Research Committee, Tabriz University of Medical Science, Tabriz, Iran.; ^3^Drug Applied Research Center and Faculty of Pharmacy, Tabriz University of Medical Sciences, Tabriz, Iran.; ^4^Research Center for Pharmaceutical Nanotechnology, Tabriz University of Medical Science, Tabriz, Iran.; ^5^Department of Mechanical Engineering, Tabriz Branch, Islamic Azad University, Tabriz, Iran.

**Keywords:** Penta block, Copolymer, Thermosensetive, Micelle, Self-assembled, Ocular

## Abstract

Many studies have focused on how drugs are formulated in the sol state at room temperature leading to the formation of in situ gel at eye temperature to provide a controlled drug release. Stimuli-responsive block copolymer hydrogels possess several advantages including uncomplicated drug formulation and ease of application, no organic solvent, protective environment for drugs, site-specificity, prolonged and localized drug delivery, lower systemic toxicity, and capability to deliver both hydrophobic and hydrophilic drugs. Self-assembling block copolymers (such as diblock, triblock, and pentablock copolymers) with large solubility variation between hydrophilic and hydrophobic segments are capable of making temperature-dependent micellar assembles, and with further increase in the temperature, of jellifying due to micellar aggregation. In general, molecular weight, hydrophobicity, and block arrangement have a significant effect on polymer crystallinity, micelle size, and in vitro drug release profile. The limitations of creature triblock copolymers as initial burst release can be largely avoided using micelles made of pentablock copolymers. Moreover, formulations based on pentablock copolymers can sustain drug release for a longer time. The present study aims to provide a concise overview of the initial and recent progresses in the design of hydrogel-based ocular drug delivery systems.

## Introduction

Of the various routes of drug delivery, ocular drug delivery is one of the most challenging ones.^1^ The complicated anatomy, physiology, and biochemistry of the eye make this organ almost impermeable to foreign substances.^2^ In order to attain an effective treatment, a sufficient quantity of active ingredient needs to be rendered and retained within the eye. Commonly used dosage forms, i.e. eye solutions, ointments, gels, and suspensions, have some drawbacks that might lead to poor ophthalmic bioavailability.^1^ Currently, there are several recommended noninvasive methods involving the use of hydrogels^3^ to increase ophthalmic bioavailability of drugs. Hydrogels are specific categories of polymeric networks that can soak up and retain a considerable amount of water while keeping their three-dimensional wholeness.^4^ Hydrogels applied for drug delivery‏ purposes are normally made ex vivo and then saturated with drugs prior to placing the hydrogel-drug complex into the body.^5^

Hydrogels can be formed using a wide variety of cross-linking techniques containing UV-photopolymerization and different chemical cross-linking procedures. Such cross-linking manners are beneficial only when the poisonous reagents are removed thoroughly before entering the hydrogel into the body. The concurrent leaching of the entrapped drug out of the hydrogel may occur during the removal of these reagents.^6^ The major shortcoming of such an approach is the necessity of the emplacement of the preformed material. Bulk hydrogels have distinct dimensions and are often highly elastic. These properties prevent their extrusion via a needle.^7^ The second‏ problem may sometimes be surpassed by turning the premade gel into micro or nanoparticles.^5^ Hydrogels may also be formed in situ in some applications, although in these cases the possible dangers of being exposed to UV radiance or to chemicals used for cross-linking has to be checked. The later problem can be overcome using the non-cross-linked linear polymers as vehicles for drug delivery.^5^ Generally, the rate of drug release from these polymers is inversely related to the viscosity of the polymer matrix.^8^ However, it seems difficult, or even infeasible, to dissolve the polymer of choice at a sufficient amount, and thereby adjust the rate of drug release to the desired limit.^5^ Even if that were feasible, the high yield stress‏ or high viscosity of the resulting substance may prevent injection or its flow through a lanky needle.^9^ Furthermore, extremely hydrophilic polymers swell in the aqueous environment inside the body and then dissolve, sometimes in a short time frame, unless they are partially cross-linked.^10^ These observations have added to the interest in formulations that display the characteristics of linear polymer solutions outside the body (letting facile injection)‏ but convert to gel upon entering inside the body (giving a long-term drug release profile).^11^ The objective of this review is to give a brief introduction to stimuli-responsive hydrogels and particularly thermosensitive micelles as drug delivery vehicles. Additionally, the most recent works on ocular drug delivery using novel pentablock copolymers are discussed at the end of the review.

### In Situ Gelling Systems

In situ (e.g. in the eye cul-de-sac) gel formation theory was first suggested in the early 1980s.^12^ In situ gel-forming formulations have the potential to be administered in liquid phase into the eye and then change into viscoelastic gel upon administration.^13^ Changes are made to the pH, temperature, and electrolyte compositions to make phase transition on the surface (Table 1).^14^

Since it is aqueous-based, the resulting swollen hydrogel is very convenient in the human eye.^15,16^ An in situ gel-forming formulation has to be a low-viscose, free-flowing liquid to be easily administered into the eye as a drop, and the gel made following the phase transition needs to be strong enough to endure the shear forces existent in the cul-de-sac and display high retention time in the eye.^17^

**Table 1:** Classification of in situ gel-forming systems

**In-situ gelling systems**	**Polymers used**
Temperature dependent systems	Chitosan, pluronics, tetronics, xyloglucans, hydroxypropylmethyl cellulose or hypermellose (HPMC)
pH-triggered systems	Cellulose acetate phthalate (CAP) latex, carbopol, polymethacrilic acid (PMMA), polyethylene glycol (PEG), pseudolatexes
Ion-activated systems (osmotically induced gelation)	Gelrite, gellan, hyaluronic acid, alginates

### Advantages of ophthalmic in situ hydrogel

The advantages of ophthalmic in situ hydrogels would be:

reduced dose concentration and frequency, improved patient compliance, ease of application in comparison with soluble or insoluble insertions, possibility of administration of exact amount of medication, dose reproducibility, and enhanced bioavailability owing to both improved pre-corneal retention time and reduced nasolacrimal drainage of the drug.^18,19^

### pH-sensitive hydrogels

pH-sensitive polymers include pendant alkaline or acidic groups that receive or release protons due to the changes in the pH of medium. The polymers with lots of ionizable groups are called polyelectrolytes.^20^ Polymers containing anionic (weakly acidic) and cationic (weakly basic) groups , respectively swell and shrink in response to increases in the external pH.^21^

### Ion-sensitive hydrogels

Ion-stimuli polymers concern the generally applied in situ gelling materials for ophthalmic drug delivery.^1^ The instilled solution changes into gel due to a change in the ionic strength. The rate of electrolyte absorption by the polymer from the tear fluid depends on the osmotic gradient across the gel surface. Therefore, the rate of sol transition into the gel is probably influenced by the osmolality of the solution. The electrolytes naturally found in the tear fluid, particularly Ca, Na, and Mg cations, induce polymers to form a gel when it is applied as a flowing solution into the cul-de-sac.^2^

### Temperature-sensitive hydrogels

Temperature-sensitive hydrogels are a group of polymeric systems that are sensitive to environmental factors. These hydrogels can swell or shrink in response to any changes in the surrounding liquid temperature.^22^ For simplicity, temperature‏-sensitive hydrogels have been categorized into three classes—positively thermosensitive, negatively thermosensitive, and thermally reversible gels.^23^

### Negatively thermosensitive hydrogels

Negatively thermosensitive hydrogels, having a lower critical solution temperature (LCST), collapse or shrink upon an increase in temperature above the LCST and swell upon a decrease in temperature below the LCST.^24,25^ Copolymers of N-isopropylacrylamide (NIAAm) display an ‘on/off’ drug release^26^ with the ‘on’ state at a lower and the ‘off’ state at a higher temperature than LCST, and give a pulsatile scheme to drug release.^27^ Generally, LCST systems are utilized to control the release of drugs, particularly proteins.^28,29^ Liposomes that thermosensitive polymers have stabilized on their membrane can release their content in a controlled manner.^30^ Bulmus *et al.* utilized PNIPAAm polymers, conjugated to a particular site near the biotin-binding site of streptavidin, for ‘on/off’ control of biotin access to its binding site.^31^ Below the LCST, i.e. 32°C for PNIPAAm, the polymer is in its completely extended conformation due to desired interaction with water molecules. In this conformation, the biotin-binding site on streptavidin is accessible to interact with biotin. However, above this temperature, the polymer collapses, preventing biotin accessibility to its binding site.^32^

### Positively thermosensitive hydrogels

Positively thermosensitive hydrogels, having an upper critical solution temperature (UCST), collapse or shrink upon a decrease in the temperature below the UCST and swell upon an increase in the temperature above the UCST.^23,33^ Polymer lattice of polyacrylamide (PAAm)^1^, poly (acrylamide-co-butyl methacrylate),^34^ and poly (acrylic acid) (PAA)^23,35^ possess positive thermosensitivity of swelling. The transition temperature of P(AAm-co-BMA) shifts to a higher temperature with increasing butyl-methacrylate content of copolymer.^32^ Aoki *et al.* fabricated an UCST system using Poly(N,N-dimethylacrylamide) combined with Poly(acrylic acid).^36^

### Thermally reversible gels

Most of the currently applied thermoreversible gels are produced by poly (ethylene oxide)-b-poly (provpylene oxide)-b-poly (ethylene oxide) (Tetronics®, Pluronics®, poloxamer).^37^ These polymers make a free-flowing solution at room temperature that can be converted to gel at body temperature.^38^ Such a system can be conveniently‏ injected into the body cavities.^39^ In some cases when decreasing the amount of the thermogelling polymer is cost effective or necessary, it can be possible to decrease the total amount of thermogelling polymer by mixing with a reversible gel-induced polymer that is sensitive to pH.^1,16,28^ New classes of biodegradable triblock copolymers have been developed. The polymers containing poly (ethylene glycol)-poly-(D‏-L‏ lactic acid-co-glycolic acid)-poly(ethylen glycol) (PEG-PLGA- PEG)^40^ or PLGA-PEG-PLGA^41,42^ were studied as injectable sustained drug delivery systems. Certain natural polymers such as xyloglucan can also be used in the formation of thermoreversible gels.^43^

### Mechanisms of gelation

To explain the sol-gel phase transition after an increase in the temperature, three main mechanisms have been suggested—gradually losing the water of hydration (desolvation‏( of the polymer, enhancing micellar accumulation, and enhancing entanglement of the polymeric lattice.^12,44^

### Micelles as thermogelling polymeric systems

**Figure 1:**
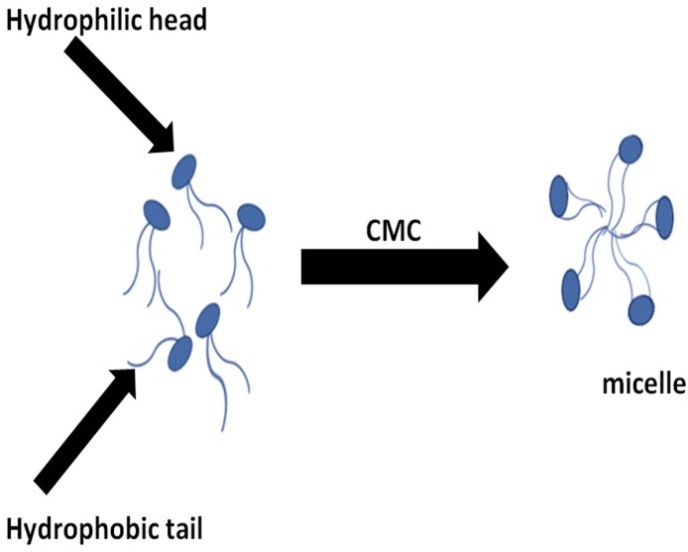
Schematic representation of micelle formation from an amphiphilic polymer

Amphiphilic block copolymers form nano-sized core-shell structures in an aqueous solution, via spontaneously self-assembling procedure,^45^ whereas polymeric micelles are connected with colloids; they are the same in certain respects to usual surfactant micelles^46^ (Figure 1). Both block copolymers and low molecular weight surfactants make micellar assemblies at or above a certain threshold called the critical micelle concentration (CMC) or the critical aggregation concentration (CAC). At a concentration less than the CMC, the number of amphiphilic molecules adsorbed at the air and water interface increases with increasing concentration. At the CMC, either the bulk solution or the interface gets saturated by unimers, while chain association occurs through the expulsion of arranged water molecules to the bulk solution.^47^

### Structure of micelles 

As regards the characteristics of micelles based on amphiphilic block copolymers, they are the ideal nominees for loading and delivery of hydrophobic drugs. Amphiphilic copolymers are composed of at least two parts that are chemically different. Thus, in solvents that selectively dissolve one of the blocks, they undergo phase dissociation because of the chain assembling.^47^ Such amphiphiles are soluble in water at low temperatures. Nevertheless, when the temperature rises, hydrophobic parts begin to assemble in order to minimize their exposure to the water molecules and thus to maximize the solvent entropy.^32^ This phenomenon resulted in the formation of a core/shell micelle structure. Theoretically, decreasing of system free energy triggers the formation of micelles. Removing hydrophobic segments from the aqueous milieu and restoring the network of hydrogen bonds in the water decrease free energy of the whole system, ultimately leading to formation of micelles.^48^

Typically, hydrophobic parts of the block copolymers form internal core of the polymeric micelles via hydrophobic interaction^48^ or through hydrogen binding,^49-51^ as well as through metal-ligand matching interactions. Moreover, there are some reports of formation of micelles via electrostatic interactions, using block copolymers of oppositely charged macromolecules, leading to the development of polyion complex(PIC) micelles.^52,53^ The hydrophilic parts of block copolymers form the external shell of polymeric micelles and play a significant role in their in vivo behaviour, particularly for their steric consolidation and the capability to interplay with cells.^54^ The conformation of polymer in solution‏ is affected by the lengths of the hydrophilic and hydrophobic segments, so that longer hydrophilic blocks cause polymers to keep in a monomeric state in water.^55^

### Characterization of micelles 

Micelles are determined by measuring the turbidity, particle size, and CMC. Ionic micellar dispersals become turbid at a higher temperature than nonionics do. The clouding aspect is an undeviating consequence of the formation of larger particles.^56^ Dynamic light scattering (DLS) is the widely used method for determination of the hydrodynamic diameter of polymeric micelles.^57,58^ Different types of methods like conductivity, interfacial tension, and osmotic pressure are utilized for the assessment of CMC.^59^ However, since the CMC values of polymeric micelles are very low, these techniques may not be useful in these cases. Light scattering is a powerful technique; however, it can be applied to portend the outset of micellization, only if the CMC happens in a range of concentrations that this method is sensitive to.^60^ Adsorption of polymer in the column is one of the problems that restrict the use of gel permeation chromatography (GPC) in determining CMC.^61^ One of the best choices for the assessment of CMC in polymeric micelles is pyrene fluorescence. The fluorescence spectrum of pyrene display particular bands near 370–400 nm, whose relative and absolute intensities, positions, and widths are highly dependent on the polarity of its microenvironment.^62,63^ Following the increase in polymer concentration, the intensity ratio of the first and third bands (I/III ratio) decreases tremendously due to changes in the polarity of pyrene environment.^64,65^ This reduction occurs owing to the accumulation of pyrene as a hydrophobic probe in the apolar micellar core around the CMC.^66^ Hence, we can easily determine the CMC by plotting the I_I_/I_III_ ratio against polymer concentration. The junction of the slope tangent and the lower horizontal is known as the CMC of the system.^67^

### Methods of drug loading into the micelles

**Figure 2:**
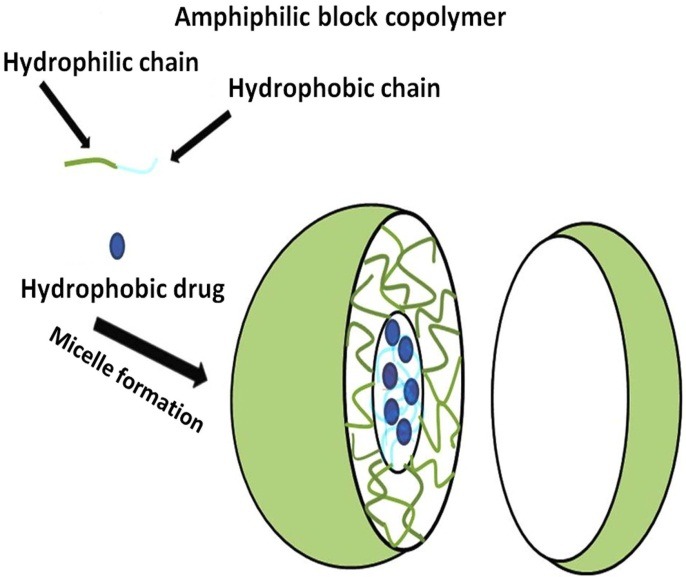
Schematic representation of incorporation of hydrophobic drug into the micelle core in an aqueous medium.

Drugs can be loaded into the micelles in physical, chemical, or electrostatic ways. However, the most preferred procedures are physical methods^68^ (Figure 2). Dialysis,^69^ direct dissolution,^70^ oil-in-water emulsion solvent evaporation,^48^ and various film-hydration methods^71^ are commonly used physical methods. Encapsulation of drug may happen within or following micelle self-assembling depending on the used method.^47,72^ In the dialysis method, both polymer and drug are dissolved in an organic solvent that is water-miscible, and then the prepared solution dialysis against a large volume of a solvent which is selective for the hydrophilic portion of copolymer.^72^ The size, polydispersity, and the yield of the polymeric micelles achieved may differ depending on the applied organic solvent.^73^ However, it is not a suitable method for industrial use due to the number days that is needed to ensure the complete removal of the applied organic solvent.^68^ In the oil-in-water emulsion method, the copolymer and drug solution are prepared in an aqueous and a volatile water-immiscible solvent, respectively. The oil-in-water emulsion is prepared by adding the organic phase containing the drug into the aqueous phase containing the copolymer and then by allowing the organic solvent to evaporate.^72^ This is not a‏ suitable method of preparing the micelles for ocular drug delivery because the complete removal of the organic solvent by evaporation is almost infeasible.^68^ As mentioned before, another method of drug loading into the micelles is the direct dissolution method. This method involves dissolving the drug and copolymer in an aqueous medium. The micelles are formed during the equilibration of the system.^68^ This method is the most convenient way of preparing micelles and is good for industrial application. However, it may not yield high amounts of drug loading.^68^ The thin-film hydration method consists of the preparation of an organic solution containing both drug and copolymer in a vial. The evaporation of the organic solvent leads to the formation of a copolymer-drug matrix film. Micelles are prepared through the rehydration of the dried film via the addition of an aqueous solvent.^72,73^ Owing to the near-complete removal of the organic solvent, this method is appropriate for the preparation of micellar ocular delivery formulations. Using this method,‏ the amount of drug loading can be significantly enhanced.^68^

Kim *et al.* reported the development of thermosensitive biodegradable hydrogels that assemble and form gels through the mechanism of micelle accumulation.^74^

These polymers can form temperature-induced micellar aggregates, and after more increasing in temperature, gels because of micellar packing.^75^ Therefore, the drugs can be mixed with these polymers at ambient temperature, in the sol state. This solution can then be administered into a target tissue where it can form an in situ gel at body temperature and control the drug release.^76^ It is a formulation that is in an injectable liquid form at ambient temperature but converts into a gel at body temperature and at a pH close to neutral. Besides, it is biocompatible and biodegradable, and certainly represents a perfect system.^38^ The temperature at which gelation takes place is affected by the chemical structure of the polymer, polymer concentration, and the length of the hydrophobic moiety.^32^ The more hydrophobic the chain, the more the driving force for hydrophobic accumulation, and the less the temperature of gelation.^5^ Mucoadhesivity of micelles can be improved by incorporating the functional groups, which are capable of binding to the chemical groups present within mucosa.^77^ Thiol is a good example of these functional groups that can interact with cysteine that is available in abundance in the mucin layer.^78^ Therefore, materials containing thiol groups can be easily attached to the mucin‏ layer and thus enhance the residence time.^79,80^

### Novel pentablock copolymers (PBCs) for sustained ocular drug delivery

In recent years, many researchers have investigated the use of nanoparticles in ocular drug delivery.^81,82^ Biodegradable polymers including poly(DL-glycolide-co-lactide) (PLGA),^83^ poly(caprolactone) (PCL),^84^ and poly(lactide) (PLA)^85^ have been extensively considered for the provision of nanoparticles. In particular, amphiphilic copolymers with polyethylene glycol as their hydrophilic segment such as PCL–PEG,^86^ PLGA–PEG,^87^ PLA–PEG,^88^ and PCL–PEG–PCL^89^ have been considered in controlled drug delivery. PEG is well known due to its nontoxicity and absence of antigenicity.^90^ Furthermore, PEG mediates the drug release via a diffusion mechanism by facilitating the penetration of water into nanoparticles. PCL is an FDA-approved, biodegradable, and nontoxic polyester that is miscible with a variety of polymers and has high permeability to small drug molecules.^91,92^ In addition, due to its hydrophobic nature, it is very capable of encapsulating lipophilic drugs through hydrophobic interactions. However, its application is limited because of its high crystallinity and hydrophobic nature,‏ which results in a very slow degradation rate.^93^ PCL- and PEG-based triblock copolymers such as PEG–PCL–PEG or PCL–PEG–PCL have been extensively studied for drug delivery. The releasing profile of the drug is greatly sustained by increasing the molecular weight of PCL block. However, high molecular weight PCL block enhances the total hydrophobicity and crystallinity of the polymer, thereby causing the initial burst release of the nanoparticles made from such triblock copolymers.^89,94^ Hence, there is still a need for optimized block copolymers that can sustain drug release over a longer time without significant initial burst release.

Patel *et al*. studied the injectable and biodegradable thermosensitive in situ gels for sustaining delivery of protein drugs in the treatment of ophthalmic posterior disease. They synthetized a series of triblock (TB) and pentablock copolymers (PBCs) of PCL-PEG-PCL, PLA-PCL-PEG-PCL-PLA and PEG-PCL-PLA-PCL-PEG, and investigated the effects of hydrophobicity, block arrangement, and molecular weight on the crystallinity of copolymer. Results of sol gel transition studies confirmed that aqueous solutions of block copolymers can convert to gel upon exposure to body temperature. Although both tri and pentablock copolymers could prolong the release of IgG, it was significantly longer for pentablock copolymers. Furthermore, the syringeability of PEG-PCL-PLA-PCL-PEG pentablock copolymer was better than both PCL-PEG-PCL and PLA-PCL-PEG-PCL-PLA copolymers due to the lower kinematic viscosity of its aqueous solution at 25°C. The crystallinity of both PBCs were lower than TBC because of the presence of PLA blocks, and therefore, it was expected that the rate of degradation of PBCs would be faster than that of TB copolymer.^95^

They also synthetized and evaluated a PB copolymer comprising PEG, polyglycolic acid (PGA), PCL, and PLA for controlled delivery of FITC-BSA, IgG, and bevacizumab in the treatment of posterior eye diseases. They studied the effect of different ratios and various molecular weights of blocks on the release profile. They showed that both the hydrophobicity of the copolymer and the hydrodynamic diameter of the loaded protein have a momentous effect on EE (entrapment efficiency) and release profile. Their studies also demonstrated that, while the nanoparticles display sustained release profile with an initial burst release, it is possible to reach a near zero order pattern of release with no or slight burst release by suspending NPs in a thermosensitive gel.^96^

In another study, they designed a series of PBCs based on PGA-PCL-PEG-PCL-PGA and PLA-PCL-PEG-PCL-PLA for sustaining delivery of IgG as a model protein. They studied the effect of polymer composition, molecular weight and isomerism on drug loading (DL), entrapment efficiency (EE), and in vitro release profile. Molecular weight and the crystallinity of copolymers indicated a considerable effect on these parameters. They moderated the crystallinity of PBCs by altering the ratios of PLA/PCL or PGA/PCL blocks, besides using different isomers of PLA (L or D,L). PBCs consisted of PLA, with D,L-lactide displaying higher EE and slower release profile compared to PB copolymers comprising PLA with L-lactide or PGA.^97^

They also synthetized a series of PBCs using PCL, PEG, and PLA or PGA, and entrapped various proteins/peptides into the prepared copolymers though the double emulsion solvent evaporation method. In order to reach a constant zero order release profile and decrease the burst release to the lowest amount, they used a novel composite conception by suspending the protein/peptide-loaded PB nanoparticles in thermosensitive PB gel. The authors investigated the influences of various parameters on DL, EE, and in vitro release profile. The results showed that an increase in molecular weight of copolymer,‏ as well as a decrease in the volume of external phase, would enhance both DL and EE. However, the addition of salt either in the external or internal phase had a small effect on EE. Besides, while there was a direct proportion between molecular weight/hydrodynamic diameter of biotherapeutics and the resulted DL or EE, the *in vitro* release rate was inversely proportional to these parameters.^98^

Tamboli *et al.* synthetized a PBC comprising PLA-PCL-PEG-PCL-PLA for sustaining the release of steroids over a longer time interval. They investigated the effect of incorporation of poly (L-lactide) (PLLA) or poly (D, L-lactide) (PDLLA) on the crystallinity of PBCs and the in vitro release profile of triamcinolone acetonide as a model drug from nanoparticles. The results showed that the incorporation of suitable ratio of PDLLA in the existent PCL-PEG-PCL copolymers lowered the crystallinity of copolymer and considerably minimized the initial burst release from NPs. The authors suggested that nanoparticles made from PBCs can minimize the limitations of‏ TBC nanoparticles such as initial burst release and can sustain the release of drug for a longer time.^93^

Khurana *et al.* designed a pentablock copolymer, PLA–PCL–PEG–PCL–PLA, to develop pazopanib-loaded nanoparticles for use in the treatment of ocular neovascularization. They studied the effect of incorporation of pazopanib (a substrate of efflux transporters) in nanoparticles on bypassing the drug efflux system .The prepared nanoparticles prolonged the delivery of pazopanib by up to 100 days without any remarkable burst release and succeeded in evading the efflux transporters^99^

Recently, Agrahari *et al.* have published their research on developing a PB copolymer composite comprising PCL-PLA-PEG-PLA-PCL IgG-Fab-loaded NPs suspended in thermosensitive mPEG-PCL-PLA-PCL-PEGm gel. Using this composite formulation, they could sustain the release of macromolecules over 80 days with negligible initial burst release occurrence. The size of the prepared NPs was 150 nm and % EE and % Dl were 66.64% ± 1.75 and 18.17% ± 0.39, respectively. The biocompatibility studies implemented on ocular (human corneal epithelial and retinal pigment epithelium) and macrophage (RAW 264.7) cell lines indicated the safety of the PB copolymer-based composite formulations for clinical uses.^100^

## Conclusion

In situ gel-forming systems are potential ocular delivery systems as they can overcome the shortcomings associated with common ocular dosage forms. Therefore, they have received much attention in recent years. Drug-incorporated liposomes, nanoparticles, micelles, etc., can also be suspended in these systems to achieve highly effective and sustained drug delivery. The limitations of available triblock polymers such as initial burst release can be largely avoided by using micelles made of pentablock copolymers. In addition, formulations based on pentablock copolymers can sustain drug release for a longer time. Thus, novel pentablock copolymers are good materials that may be used as a carrier for ophthalmic drug delivery as well as for other illnesses that need sustained drug delivery.

## Acknowledgments

The financial support from Drug Applied Research Center and Research Council of Tabriz University of Medical Sciences is greatly acknowledged.

## Ethical Issues

Not applicable.

## Conflict of Interest

The authors have no conflicts of interest to declare.
